# Deciphering the Molecular Mechanism Underlying the Inhibitory Efficacy of Taiwanese Local Pomegranate Peels against Urinary Bladder Urothelial Carcinoma

**DOI:** 10.3390/nu10050543

**Published:** 2018-04-27

**Authors:** Ching-Ping Chang, Yu-Yi Chan, Chien-Feng Li, Lan-Hsiang Chien, Song-Tay Lee, Ting-Feng Wu

**Affiliations:** 1 Department of Medical Research, Chi Mei Medical Center, Tainan 710, Taiwan; jessica.cpchang@gmail.com (C.-P.C.); m96h0207@stust.edu.tw (L.-H.C.); 2 Department of Biotechnology, Southern Taiwan University of Science and Technology, Tainan 710, Taiwan; yuyichan@stust.edu.tw (Y.-Y.C.); angelo.p@yahoo.com.tw (C.-F.L.); songtlee@stust.edu.tw (S.-T.L.); 3 Department of Pathology, Chi Mei Medical Center, Tainan 710, Taiwan

**Keywords:** apoptosis, T24 cells, death receptor pathway, mitochondrial pathway, endoplasmic reticulum stress, pomegranate peel, urinary bladder urothelial carcinoma

## Abstract

Pomegranate (*Punica granatum* L.) fruit has been demonstrated to have the inhibitory activities to various tumors. In this study, we try to uncover the molecular mechanism underlying the inhibitory capability of Taiwanese local pomegranate fruit to urinary bladder urothelial carcinoma. The results collected from the 3-(4,5-dimethylthiazol-2-yl)-2,5-diphenyltetrazolium bromide assay indicated that the ethanol extract of pomegranate peel exhibited better inhibitory activity to human urinary bladder urothelial carcinoma T24 and J82 cells than that of pulp. Furthermore, the ethylacetate layer of peel ethanol extract was observed to have the best inhibitory activity against urinary bladder urothelial carcinoma cells. One of the eight fractions (PEPE2 fraction) collected from the ethylacetate layer with Diaion HP-20 column chromatography demonstrated the highest inhibitory activity in urinary bladder urothelial carcinoma cells. The results of the flow cytometry and apoptotic pathway studies suggested that the inhibitory activity of PEPE2 fraction were attributed to the UBUC cell apoptosis. To confirm the above results, our results of xenograft-induced bladder tumor in nude mice showed that the oral consumption of the ethylacetate layer (2, 5, 10 and 100 mg/kg) could decrease the volume and weight of T24 tumors and caused the apoptosis in the xenografted tumors, which was observed by terminal deoxynucleotidyl transferase-mediated deoxyuridine triphosphate nick end-labeling assay. This study provided the likelihood that the traditionally non-edible pomegranate peel waste is re-utilized to make an affordable and promising chemopreventive product to prevent UBUC incidence or recurrence.

## 1. Introduction

Bladder cancer is the most common tumor of the urinary system in the world and ranked the 9th in male cancer incidence in Taiwan in 2014 [1,2]. Urothelial carcinoma is the most encountered lesion among various bladder tumor types, which contributes to more than 90% of the bladder cancer cases in developed countries [1]. According to WHO classification (2004), urinary bladder urothelial carcinoma (UBUC) cells can be classified into the low or high grades. Most of the UBUCs are papillary/non-invasive or superficially invasive types and can be treated by curettage. However, local recurrence can be observed in some UBUCs even followed by lethal distal spreading [3]. In terms of disease management, it may be beneficial that a chemopreventive product for bladder cancer can be used by cancer patients or high-risk persons to avoid tumor recurrence or incidence.

Pomegranate (*Punica granatum* L.) is an edible fruit collected from a deciduous tree species. Today, it is widely grown in Mediterranean countries, Northern India, Northern/Southern America, Europe, and even in Taiwan, mainly as gardening trees. The pomegranate fruit is observed to have a reddish peel and white to deep red seeds. Pomegranate pericarp (peel) is a rich resource of phenolics, flavonoids, ellagitannins (predominantly punicalagin), and proanthocyanins compounds [4]. Pomegranate seeds are edible and have strong anti-oxidant activities attributed to the high amount of hydrolyzable tannins and anthocyanins [4]. Pomegranate juice (PJ) squeezed from seed pulp is rich in phenolics and flavonoids, mainly anthocyanins [4].

Many studies exploring the chemopreventive capability of pomegranate have indicated that the pomegranate fruit demonstrates inhibitory activities to various tumors. Pomegranate fruit extract (PFE) prepared from the edible portions (seed coats and pulp juice) of the pomegranate fruit with 70% acetone was proved to display the apoptotic impacts on human lung cancer A549 cells but have minimal effects on normal bronchial epithelial cells [5]. PFE treatment also induces G_0_/G_1_ arrest and inhibits not only NF-κB activities but also various MAP kinase pathways [5]. PFE administration also ameliorates tumor growth/progression/angiogenesis of B(a)P or NTCU induced primary lung tumors in A/J mice by dwindled activation of NF-κB, MAP kinase pathways, and mammalian target of rapamycin (mTOR) signaling [6]. In addition to the effects on lung cancer, PFE may be a promising chemopreventive/chemotherapeutic agent against human prostate cancer as well. Malik et al. [7] found that apoptosis is evoked in PFE-treated highly aggressive PC3 cells, resulting in a decrease in Bcl-2 along with an increase in Bax. Their studies showed that PFE treatment impairs various cell cycle cyclins and p21 as well as p27. Additionally, the oral administration of 0.1% or 0.2% PFE in drinking water (equivalent to the ingredients present in 250 or 500 mL of pomegranate juice) to TRAMP mice can obviously inhibit prostate carcinogenesis by inhibiting IGF-1/Akt/mTOR pathways [8]. Retting et al. [9] reported that the treatment of polyphenols/ellagitannin-abundant extract (PE) purified from pomegranate fruit peels can restrict LAPC4 androgen-independent xenograft via the inhibition of the NF-κB signaling pathway. The clinical trial in patients with increasing prostate-specific antigens (PSA) after surgery or radiotherapy demonstrated that daily oral consumption of 8 ounces of pomegranate juice (PJ) significantly extends the PSA doubling time (PSADT) from 15 to 54 months in men with prostate cancer (PCa) [10]. However, a randomized, double-blind, placebo-controlled clinical study also showed that PFE treatment does not significantly extend the PSADT in PCa patients with rising PSA after primary therapy, compared to the placebo-treated group [11]. Our previous documented results demonstrated that Taiwanese local pomegranate juice (PJ) could evoke cell apoptosis via the intrinsic mitochondrial pathway and the extrinsic death receptor signaling in PCa cells. It also can de-regulate the expression levels of genes linked to cytoskeletal functions, anti-apoptosis, metabolism, NF-B signaling in PJ-treated PCa cells [12]. Based on the above-documented findings, pomegranates may be a promising chemopreventive or chemotherapeutic source against UBUC carcinogenesis and recurrence.

In this study, we found that the ethanol extract isolated from pomegranate peels (PEP) exhibited a better inhibitory activity comparatively to UBUC cells than that of pulps. Fractionation of PEP with increasing polarity showed that the EtOAc fraction of PEP had the best anti-cancer efficacy. Treatment of 1 of 8 column-fractionation parts of the EtOAc fraction, PEPE6, could evoke UBUC cell apoptosis. Oral administration of the EtOAc fraction of PEP to UBUC xenografted nude mice caused a significant reduction in tumor growth and evoked cell apoptosis. Taken together, our results implied that the ingredients present in pomegranate peels demonstrated the inhibitory activity of UBUC cells.

## 2. Materials and Methods

### 2.1. Materials

The following antibodies were used for western immunoblotting: Actin (1:5000, Millipore, Billerica, MA, USA), Bip (1:1000, BD Biosciences, San Jose, CA, USA), Caspase-3 (1:250, Gene Tex, Irvine, CA, USA), Caspase-8 (1:250, BD Biosciences, San Jose, CA, USA), Caspase-9 (1:1000, BD Biosciences, San Jose, CA, USA), Caspase-12 (1:1000, Gene Tex, Irvine, CA, USA), DR4 (1:1000, Gene Tex, Irvine, CA, USA), DR5 (1:2000, Gene Tex, Irvine, CA, USA), VCP (1:1000, Abnova, Taipei, Taiwan, ROC), Anti-rabbit IgG (1:10,000, GE Healthcare, Mickleton, NJ, USA), and Anti-mouse IgG (1:10,000, GE Healthcare, Mickleton, NJ, USA). Annexin V conjugated with FITC was bought from the Strong Biotech Corporation in Tainan, Taiwan. Chemiluminescence ECL detection system was purchased from GE Healthcare Bio-Sciences AB in Uppsala, Sweden. Dulbecco’s Modified Eagle Medium, McCoy’s5A, and fetal bovine serum were purchased from GIBCO, Grand Island in NY, USA. MTT was bought from Merck in Darmstadt, Germany. Propidium iodide was purchased from Sigma, Saint Louis in MO, USA. PVDF membrane was bought from Stratagene, La Jolla in CA, USA. TUNEL reaction mixture was purchased from Rochel in Mannheim, Germany.

### 2.2. Collection and Identification of Plant Materials

The fruits of the *P. granatum* were field collected from a farmland (22°41′59.3267″ N, 120°30′45.1836″ E) located in Jiuru, a suburban township in the Pingtung county in southern Taiwan from August to September 2012. The plant specimens were identified by Dr. Gwo-Ing Liao from National Chen-Kung University, Taiwan and were pressed/dried for voucher specimens (Nan-Kai Lin, STUSTG308-001 to STUSTG308-003) deposited in the herbarium of Taiwan Forestry Research Institute (TFRI), Taiwan.

### 2.3. Preparation of the Ethanol Extracts from Pulps and Peels of Pomegranates

**Figure 1:**
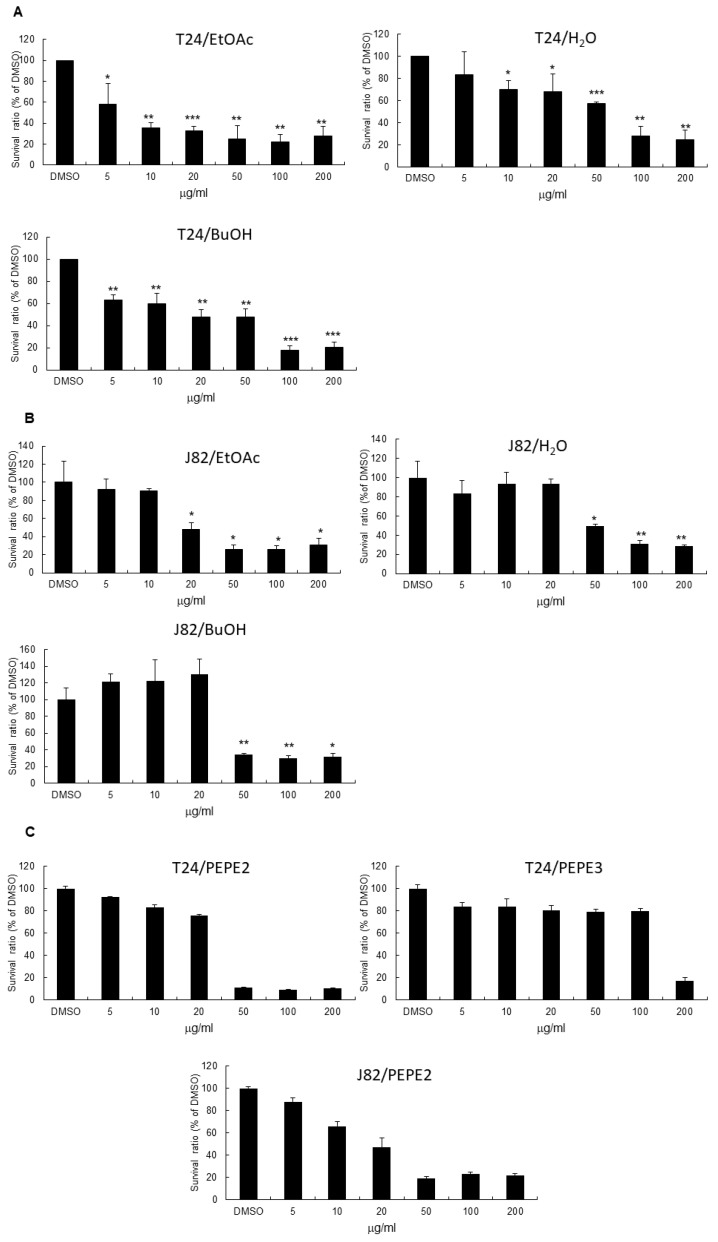
The inhibitory activities of Taiwanese local pomegranate fruit. The ethylacetate, butanol, and water layers extracted from pomegranate peel extract (PEP) were tested for the inhibitory efficacy to T24 (**A**) or J82 (**B**) cells using MTT assay as described in Materials and Methods. PEPE2 and PEPE3 (**C**) were also investigated for the inhibitory effectiveness to T24 or J82 cells. 0.1% (*v*/*v*) Dimethyl sulfoxide (DMSO)-treated urinary bladder urothelial carcinoma (UBUC) cells were regarded as the solvent control. Each MTT result was the typical data of at least three independent experiments. * *p* ≤ 0.05, ** *p* ≤ 0.01, *** *p* ≤ 0.001.

Fresh pomegranate pulps (452 g) and peels (392 g) were extracted respectively with 95% ethanol, three times, at a ratio of 1:3 at room temperature for 24 h. The filtrates were evaporated under reduced pressure to yield the dark brown syrups from pulp (PEG, 51 g) and peel (PEP, 74 g) extracts respectively. PEP was suspended in water (300 mL) and then partitioned with ethyl acetate (EtOAc) (4 × 300 mL) and *n*-butanol (4 × 300 mL) successively to yield individual layers of extracts of EtOAc (4.5 g), *n*-butanol (29.8 g) and water (28.7 g), respectively. The above three layers were examined for their anticancer bioactivities. Among these three layers, the EtOAc layer exhibited the most potent effectiveness (Figure 1). Hence, the EtOAc layer (4.5 g) was subjected directly to Diaion HP-20 column chromatography, eluted with water containing increasing proportions of ethanol to render eight fractions labeled PEPE1 (0.1256 g), PEPE2 (0.1064 g), PEPE3 (1.712 g), PEPE4 (1.4595 g), PEPE5 (0.3758 g), PEPE6 (0.1384 g), PEPE7 (0.1731 g), and PEPE8 (0.1567 g). The eight fractions were then evaluated for their anticancer bioactivities.

### 2.4. Cell Lines

Human UBUC T24 cells, identified as high grade and invasive, were purchased from the Bioresource Collection and Research Center, Hsinchu, Taiwan and cultured at 37 °C in McCoy’s5A supplemented with 10% (*v*/*v*) fetal bovine serum. Human UBUC J82 cells recognized as high grade were provided by Dr. Chien-Feng Li from Department of Pathology, Chi-Mei Medical Center, Tainan, Taiwan and maintained at 37 °C in Dulbecco’s Modified Eagle Medium supplemented with 10% (*v*/*v*) fetal bovine serum. Human papillomavirus E7 immortalized uroepithelial cell was kindly provided by Professor Hsiao-Sheng Liu from the department of microbiology and immunology, college of medicine, National Cheng Kung University, Tainan, Taiwan and maintained as described previously [13].

### 2.5. 3-(4,5-Dimethylthiazol-2-yl)-2,5-diphenyltetrazolium Bromide (MTT) Assay

As indicated in Figure 1, 5, 10, 20, 50, and 100 μg/mL of ethanol extracts of pomegranate peels were added to a 96-well plate seeded with 5000 human T24 cells, 6000 human J82 cells, or 3000 human E7 cells per well. The same concentrations and protocol were also conducted with pulp ethanol extracts and further with EtOAc, BuOH and H_2_O layers from peel ethanol extracts and PEP2/PEP3 column fractions. After incubation for the time period shown in Figure 1, 20 μL of MTT solution (5 mg/mL PBS) was added to each well and the plate was incubated at 37 °C for 4 h. After medium removal, 200 μL of dimethyl sulfoxide (DMSO) was added to each well and the plate was gently shaken for 5 min. The absorbance was measured at 540 nm. Quadruplicate wells were applied to each concentration for a specific time period. 0.1% (*v*/*v*) DMSO (vehicle)-treated UBUC cells were recognized as the control.

### 2.6. Cell Cycle Analysis of PEPE2-Treated UBUC Cells

1 × 10^6^ T24 or 7 × 10^5^ J82 cells were first seeded on a 10-cm plate. After overnight incubation, 50 μg/mL and 20 μg/mL of PEPE2 was added to respectively T24 and J82 cells seeded on each 10-cm plate and the cells were harvested at the appropriate time duration. The collected cells of each plate were suspended in 500 μL of ice-cold 70% ethanol at 4 °C overnight. After treatment, the cells were washed with 1 mL ice-cold PBS and re-suspended in 100 μL PBS. Afterward, the cells were incubated in 300 μL propidium iodide (PI) solution (3 μL RNase and 20 μg PI per mL) in the dark at 37 °C for 30 min. The stained cells were analyzed by FACSCalibur flow cytometer (Becton Dickinson, Franklin Lakes, NJ, USA). 0.1% (*v*/*v*) DMSO-treated UBUC cells were regarded as the control and analyzed as described above.

### 2.7. Annexin V/PI Analyses of PEPE2-Treated UBUC Cells

T24 and J82 cells were treated as indicated in the annexin V/PI analyses. The collected cells of each plate were washed with 1 mL ice-cold PBS and re-suspended in 100 μL binding buffer. Then 2 μL of annexin V conjugated with FITC and 2 μL of PI solution were administrated to the re-suspended cells and incubated in the dark on ice for 15 min. After incubation, the cells were measured using a FACSCalibur flow cytometer (Becton Dickinson). A percentage of 0.1% (*v*/*v*) DMSO-treated UBUC cells were deemed as the control and analyzed as described above.

### 2.8. Western Immunoblotting

After treatment, as described in the results, T24 or J82 cells were harvested and lysed in the lysis buffer (10 mMTris (pH 8.0), 0.32 M sucrose, 1% (*v*/*v*) Triton X-100, 5 mM EDTA, 2 mM DTT, and 1 mM PMSF). After determining its protein concentration using Bio-Rad DC protein assay kit, equal volume of 2× sample buffer (0.1 M Tris (pH 6.8), 2% (*w*/*v*) SDS, 0.2% (*v*/*v*) β-mercaptoethanol, 10% (*v*/*v*) glycerol, and 0.0016% (*w*/*v*) bromophenol blue) was mixed with the protein lysate. The protein lysates were separated by SDS-PAGE at 100 V for the suitable time and further transferred onto a PVDF membrane. After blocking for 1 h in 3% (*w*/*v*) bovine serum albumin at room temperature, the membranes were hybridized for 2 h at room temperature with primary antibodies. Then the membranes were washed and hybridized with correspondent secondary antibodies for 1 h at room temperature. The secondary antibodies binding on the membrane were detected by a chemiluminescence ECL detection system using Fujifilm LAS-4000 Luminescent Image Analyzer (Fujifilm Corporation, Tokyo, Japan). The intensity of each protein band of interest was quantified by PDQUEST Quantity One software (Bio-Rad Laboratory, Hercules, CA, USA) and normalized with actin protein expression level. The acquired data were analyzed with Student’s *t*-Test (STATISTICA, StatSoft, Tulsa, OK, USA). The 0.1% (*v*/*v*) of DMSO-treated UBUC cells were deemed as the control and analyzed as described above.

### 2.9. Xenografted Tumors of T24 Cells in Nude Mice

Relevant animal studies were performed in accordance with the Guide for Laboratory Animal Facilities and Care as promulgated by the Council of Agriculture, Executive Yuan, Taiwan. The protocol was approved by the Animal Research Committee (permit number: MED-100-05; project code: NSC 101-2632-B-218-001-MY3) of Southern Taiwan University of Science and Technology. Male nude mice (BALB/cAnN-Foxn1, 9 weeks old) were purchased from the animal center of the National Science Council in Taiwan. The animals were nurtured at 23–25 °C and 30–70% humidity with 12-h light/12-h darkness cycle. The mice intake rodent Lab Diet 5001 (Lab Supply, Fort Worth, TX, USA) and sterile water ad libitium. The mice were quarantined for 7 days before the experiment. The mice were subcutaneously (s.c.) inoculated on the right lower abdomen with 100 μL T24 cell/matrigel mixture prepared by blending 1 × 10^7^ of T24 cells with an equal volume of the gel. All efforts were made to minimize the suffering of the mice. Dried EtOAc extract was suspended thoroughly in water and then the suspension was used for oral gavage (o.g.). The mice were separated into 4 subgroups. The xenografted mice were fed o.g. with water (*n* = 11), 2 mg/kg (*n* = 8), 5 mg/kg (*n* = 10), 10 mg/kg (*n* = 10), and 100 mg/kg (*n* = 6) of EtOAc extract. For the extract-treated groups, after implanted with T24 cells, the mice were fed with EtOAc extract suspension by o.g. in the next day and then administrated with the suspension once a day until the endpoint. At the endpoint, each mouse was euthanized by intraperitoneal (i.p.) injecting with 0.5 mL of 500 mg/mL urethane and the tumor, as well as liver, was collected. Growth of the xenografted tumors was measured by vernier calipers at 3-day intervals. The tumor volume was calculated as V = length × width^2^/2 as described previously [14]. For Statistical Analysis, the results are expressed as the mean ± standard error (SE) of at least 3 independent experiments. Statistical significance was determined by Student’s *t*-test and one-way ANOVA using the SigmaPlot program for Windows, version 12.0 (Systat Software Inc., San Jose, CA, USA).

### 2.10. Terminal Deoxynucleotidyl Transferase-Mediated Deoxyuridine Triphosphate Nick End-Labeling Assay (TUNEL)

Histologic specimens were prepared in the way as described for preparation of the subcutaneous xenografts. Animal specimens were fixed in 10%-buffered formalin solution and embedded in paraffin. For morphological analysis, the hematoxylin-eosin (H&E) staining and microscopic examination were performed on the 3-μm-thick sections of the paraffin-embedded tumor blocks. Slides were either stained with H&E according to the aforementioned specifications or exposed to TUNEL assay. The sections were treated with xylene and then with ethanol. After paraffin removal and dehydration, the sections were washed with PBS and incubated with 3% (*v*/*v*) H_2_O_2_ solution for 20 min. Then the specimens were treated with 5 μg/mL proteinase k at room temperature for 2 min. After enzyme incubation, the specimens were washed with 0.1 M PBS (pH 7.4) and incubated with a TUNEL reaction mixture at 37 °C for 1 h. Then the treated sections were washed with distilled water and hybridized with a horse-radish peroxidase-conjugated fluorescent antibody at room temperature for 30 min. Lastly the sections were washed with distilled water. The TUNEL-positive cells were evaluated in at least 6 fields per section (×200 magnification) by two persons under a blinding condition.

### 2.11. Statistical Analyses

Student’s *t*-test (STATISTICA Ver 10.0 MR1, StatSoft, Tulsa, OK, USA) were used for the statistical analyses of MTT assay, flow cytometry, and western immunoblotting. Student’s *t*-test and one-way ANOVA of the SigmaPlot program for Windows, version 12.0 (Systat Software Inc., San Jose, CA, USA) were exploited for the statistical significance of animal studies.

## 3. Results

### 3.1. Pomegranate Fruits Exhibited the Inhibitory Impacts on UBUC Cell Proliferation

In this investigation, the pulps and peels of fresh pomegranate fruits were extracted with ethanol, respectively, to give pulp extract (PEG) and peel extract (PEP). The results of MTT assays showed that PEP exhibited better suppressive ability than that of PEG toward UBUC cells (Figure S1A,B). Therefore, PEP was partitioned successively between H_2_O/EtOAc and between H_2_O/*n*-BuOH, respectively, to yield three layers of EtOAc, *n*-BuOH, and H_2_O. The results of the MTT assays demonstrated that IC_50_ of EtOAc, butanol, and water layers from PEP were 5, 10, and 50 μg/mL, respectively, against the T24 cells while for the J82 cells IC_50_ of these three layers were 20, 50, and 50 μg/mL (Figure 1A,B). Taken together, the EtOAc layer had the best inhibitory activity against the UBUC cell lines. The HPLC profile of EtOAc layer was shown in Supplementary Figure S2A.

Furthermore, the EtOAc layer was fractionated into 8 fractions by Diaion HP-20 column chromatography as described in Materials and Methods. We first examined the toxicity of each column fraction to normal-like urothelial E7 cell and found that IC_50_ of PEPE2 and PEPE3 were >200 μg/mL, respectively, (Figure S1C) and the other PEP fractions would harm E7 cell (data not shown). Findings in Figure 1C demonstrated that the PEPE2 fraction showed the best inhibitory effects on human T24 and J82 cells and the least harmful impacts on normal-like human E7 urothelial cells. The HPLC map of PEPE2 was shown in Supplementary Figure S2B.

### 3.2. PEPE2-Evoked Retardation of Human UBUC Cell Proliferation Attributed to Cell Apoptosis

**Figure 2:**
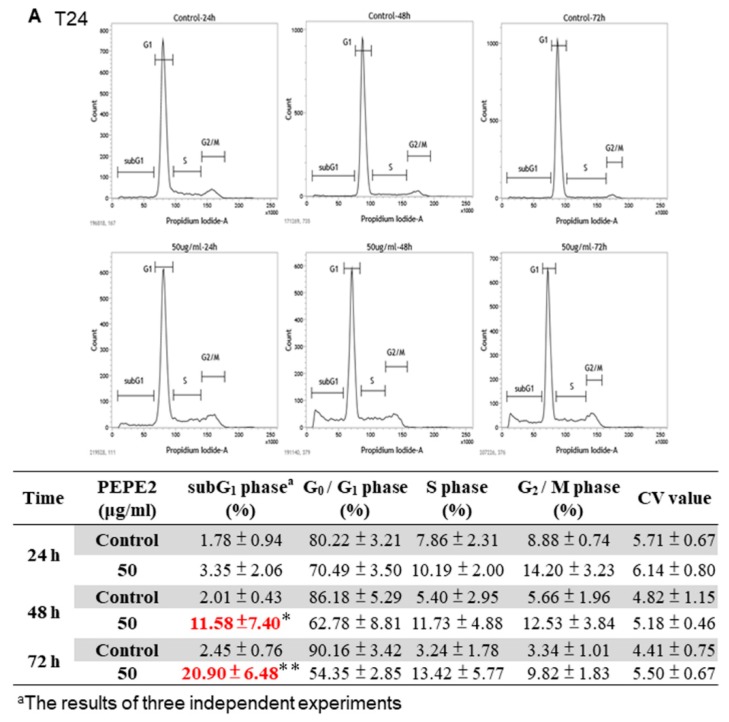

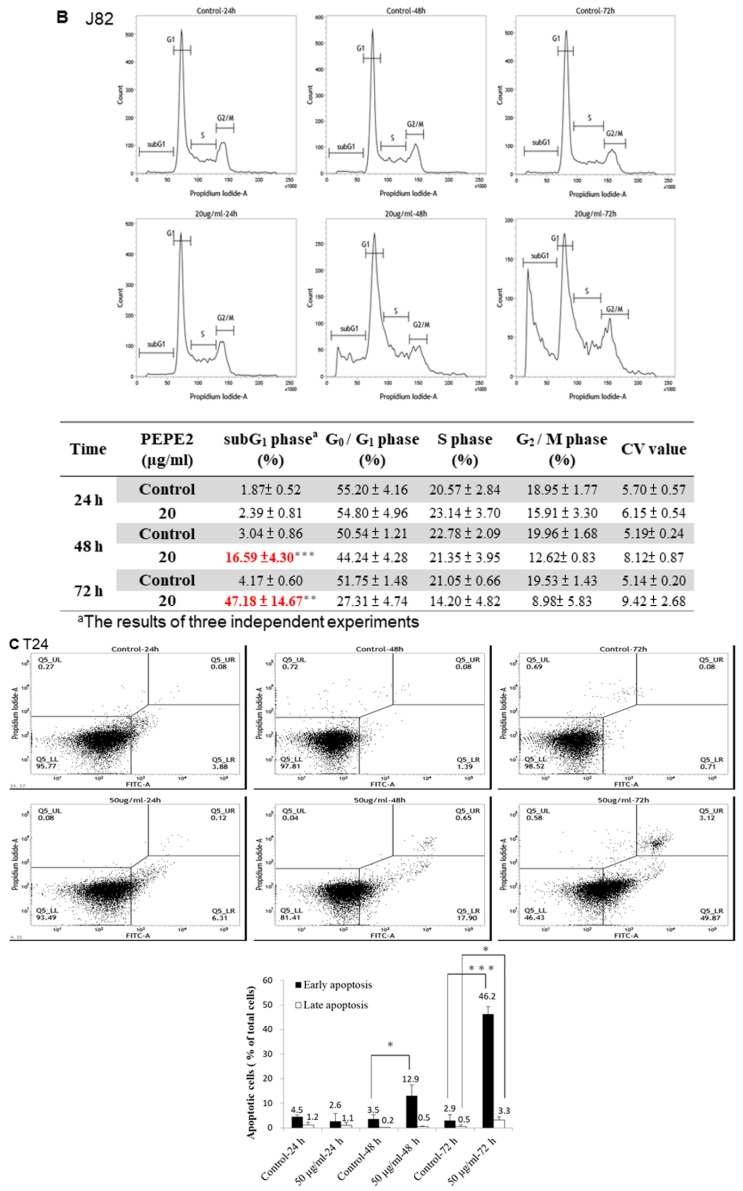

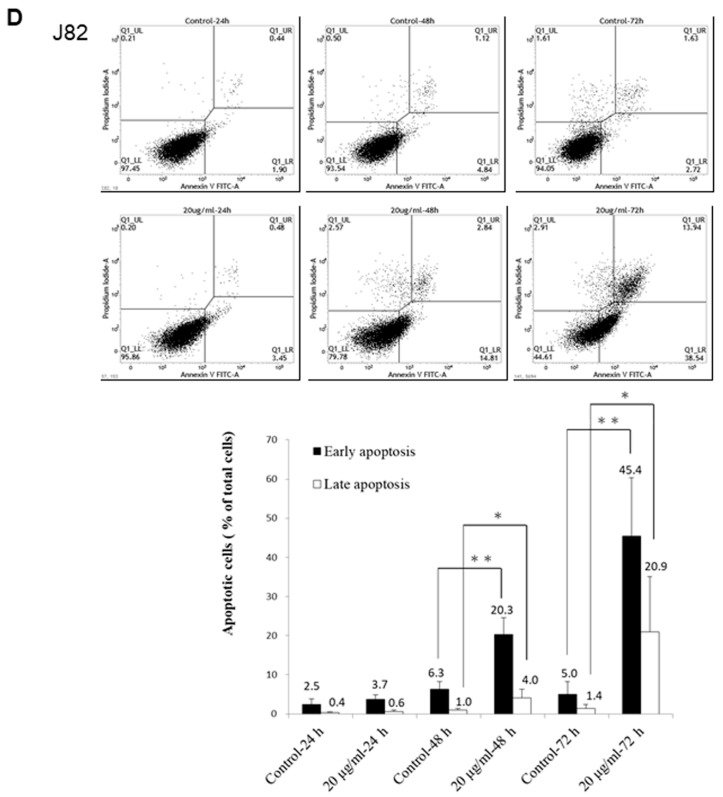
The propidium iodide (PI) and PI/annexin V analyses of UBUC cells treated with PEPE2. The results of PI analyses were represented in (**A**) the T24 cells and (**B**) the J82 cells. The data of the PI/annexin V measurement were shown in (**C**) T24 cells and (**D**) J82 cells. Each flow cytometry figure was the typical result of three independent experiments. The diagram under the PI/annexin V panel was the results of the three independent experiments. The cell cycles and apoptosis detection of the T24 or J82 cells treated with PEPE2 were measured with PI and PI/annexin V analyses, respectively, using flow cytometry as described in the supplementary document. The 0.1% (*v*/*v*) DMSO-treated UBUC cells were implemented as the vehicle control. * *p* ≤ 0.05, ** *p* ≤ 0.01, *** *p* ≤ 0.001.

To discover which mechanism was engaged in the inhibition of human UBUC cell proliferation, the cell cycle analyses conducted following incubation of T24 cells with 50 μg/mL PEPE2 showed that the PEPE2 treatment induced 20.9% on average of T24 cells in the sub-G_1_ phase at 72-h as compared to 2.45% on the average DMSO-treated cells (Figure 2A), whereas treatment with 20 μg/mL PEPE2 evoked 41.78% on average of J82 cells in the subG1 phase at 72-h as compared to 4.17% on average of DMSO-treated cells (Figure 2B). Furthermore, the annexin V/PI analysis indicated that 50 μg/mL of PEPE2 incubation for 72 h enhanced the early apoptotic T24 cells significantly from 2.9 to 46.2% on average as compared to the vehicle-treated cells (Figure 2C) while 20 μg/mL PEPE2 treatment for 72 h augmented the early/late J82 apoptotic cells prominently from 6.4 to 66.3% on average as compared to the DMSO-treated cells (Figure 2D), suggesting that the PEPE2 treatment could induce apoptosis in the T24 and J82 cells and J82 cells that were more susceptible to PEPE2 than that of T24 cells.

### 3.3. Molecular Mechanism of the Apoptotic Pathway Induced in PEPE2-Treated UBUC Cells

**Figure 3:**
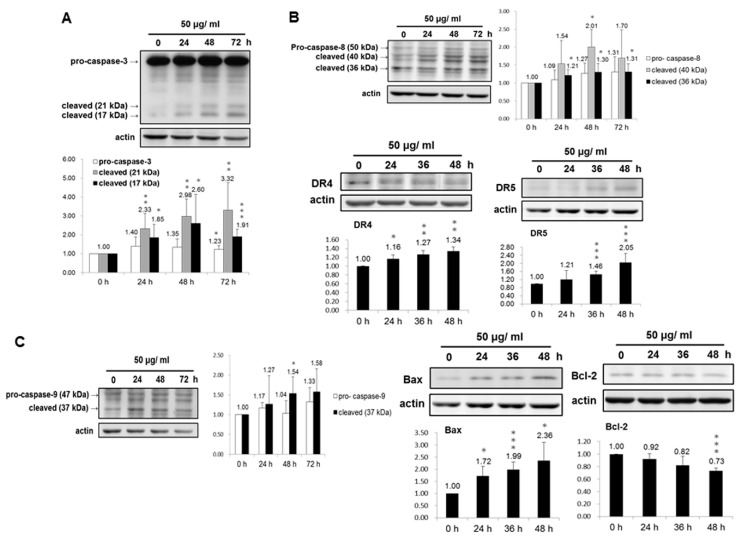

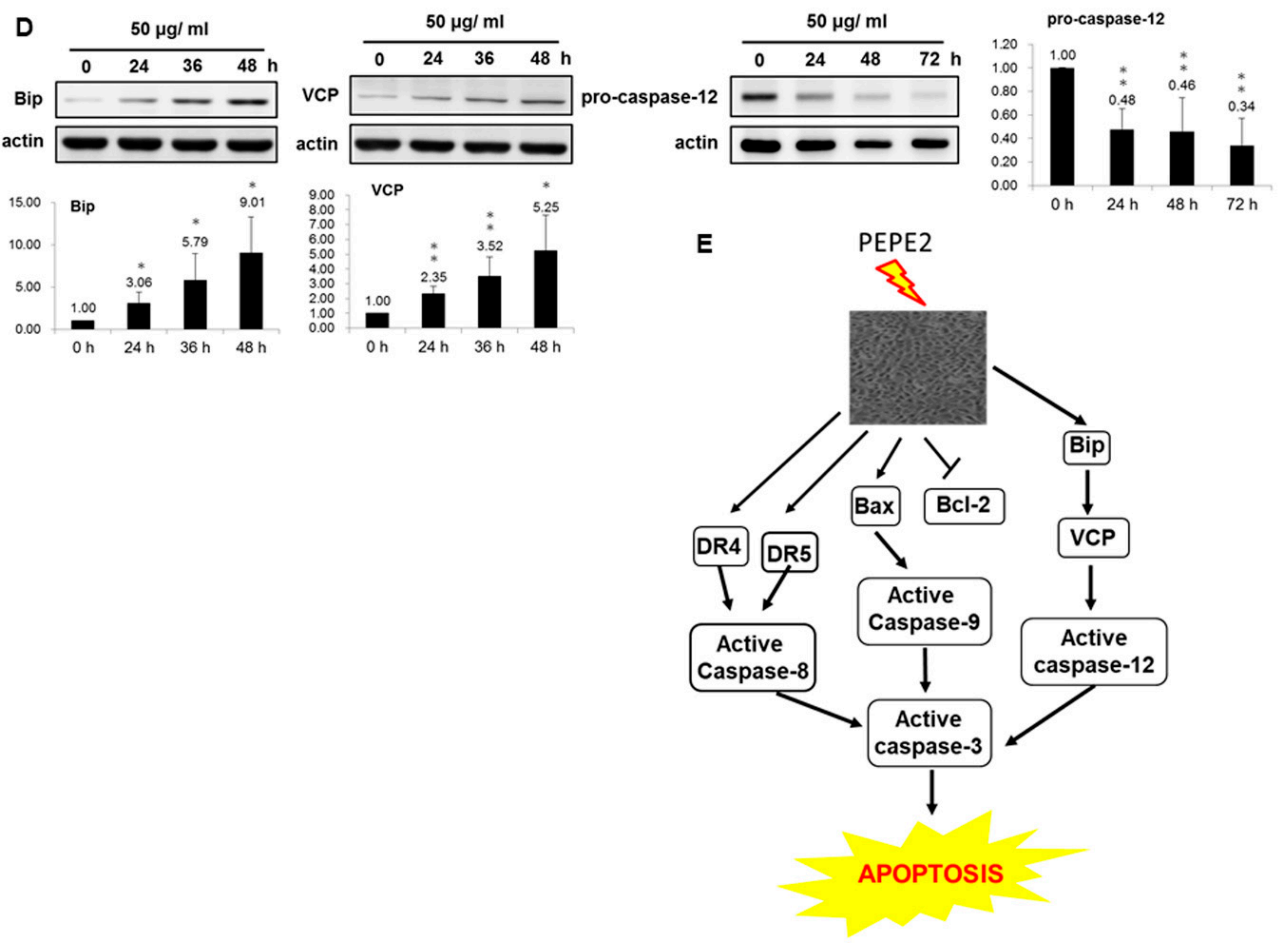
The molecular mechanisms of apoptotic pathway evoked in PEPE2-incubated UBUC cells. T24 and J82 cells were treated with 50 and 20 μg/mL PEPE2 respectively. Then the protein levels of (**A**) pro-/cleaved caspase-3; (**B**) pro-/cleaved caspase-8, DR4 and DR5; (**C**) pro-/cleaved caspase-9, Bax and Bcl-2 and (**D**) Bip, VCP and pro- caspase-12 in PEPE2-treated T24 cells were measured using western immunoblotting as described in the supporting information. The 0.1% (*v*/*v*) DMSO-treated UBUC cells were used as the solvent control; (**E**) The proposed molecular apoptotic pathway provoked in the PEPE2-treated UBUC cells. The immunoblot in each figure was the representative result of at least three independent experiments. The diagram (ratio (mean ± standard deviation (S.D.)) under each immunoblot indicated the ratio of the normalized protein intensity (observed protein/actin) of PEPE2-treated cells at the indicated time interval, divided by that at the 0-h time point. * *p* ≤ 0.05, ** *p* ≤ 0.01, *** *p* ≤ 0.001.

The previous results showed that the PEPE2 treatment could induce bladder cancer cell apoptosis. However, the cytometric analysis cannot decipher whether PEPE2 resulted in the apoptosis by death receptor signaling, the mitochondrial damage pathway, or ER stress. Caspase-3 is a key player in mitochondrial damage, death receptor signaling, and ER stress. Meanwhile, Caspase-3 can be activated by caspase-8 in death receptor signaling, by caspase-9 in mitochondrial damage, and by caspase-12 in endothelium reticulum (ER) stress [15]. In order to search for the molecular mechanism by which PEPE2 evoked the apoptosis, the processing and activation of caspase-3 was measured in drug-treated UBUC cells. The results in Figure 3A showed that the activated caspase-3 (21 and 17 kDa) amount was increased in PEPE2-treated T24 cells in a time-dependent response, implicating that caspase-3 was activated while the apoptosis might be initiated in PEPE2-incubated T24 cells. The data in Figure 3B and Figure S3A also demonstrated that the expressions of DR4 as well as DR5 and activated caspase-8 were augmented, implicating that the death receptor pathway was evoked in PEPE2-treated T24 and J82 cells. In PEPE2-incubated T24 and J82 cells, the apoptosis-activator Bax level was increased while the anti-apoptotic Bcl-2 amount was decreased and thus, the pro-caspase-9 level dwindled, implying that the mitochondrial pathway was related to cancer cell apoptosis (Figure 3C and Figure S3B). Our studies also showed that the amount of Bip and VCP (ER stress markers) was increased in PEPE2-treated T24 and J82 cells and thus, pro-caspase-12 was activated (Figure 3D and Figure S3C), implying that the ER stress was also associated with UBUC cell apoptosis.

### 3.4. The EtOAc Layer of PEP Could Reduce Xenografted Tumor Growth in Nude Mice 

**Figure 4:**
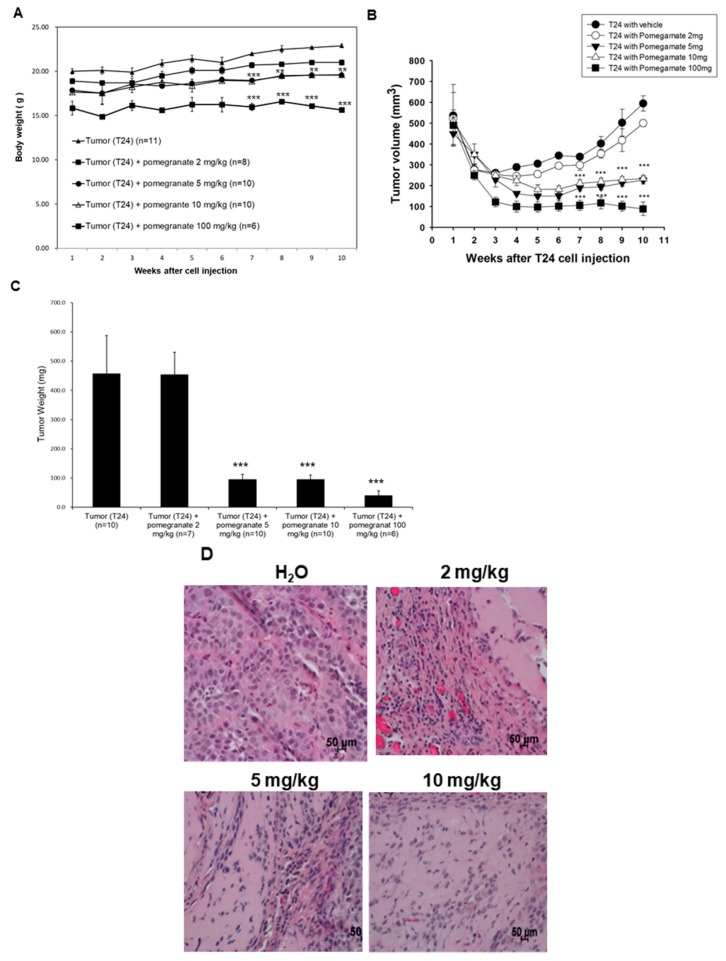

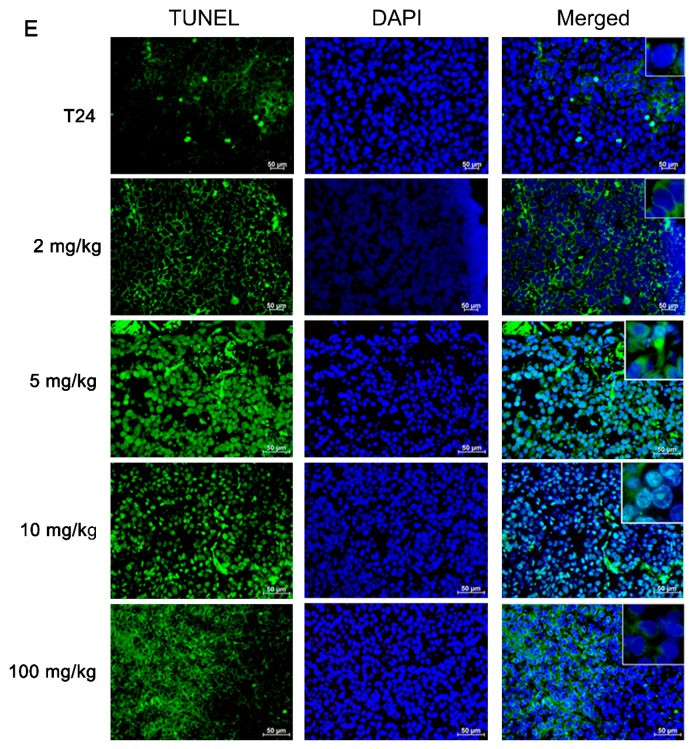
The inhibition of the xenografted UBUC growth in nude mice by the treatment of the EtOAc layer. T24 cells were injected s.c. into nude mice and were o.g. administrated with water (control), 2, 5, 10, or 100 mg/kg EtOAc layer as described in Materials and Methods. (**A**) The body weights of the extract-treated mice. *** *p* < 0.001, ** *p* < 0.005; (**B**) The effects of the EtOAc layer on tumor growth in the xenografted nude mice. The volumes of tumors from the extract-fed mice were compared to those of the water-fed mice. *** *p* < 0.001; (**C**) The tumor weight. The tumor weight was measured at the 10th week. *** *p* < 0.001; (**D**) The typical H/E images (400×) of tumor specimens dissected from xenografted nude mice; (**E**) The representative terminal deoxynucleotidyl transferase-mediated deoxyuridine triphosphate nick end-labeling (TUNEL) images (400×) of tumor lesions from xenografted nude mice. H&E and TUNEL assays were performed as described in Material and Methods.

In order to further confirm the UBUC-cell-line-associated inhibitory activity of pomegranate fruits, xenografted tumors induced by implanting T24 cells into nude mice as described in Materials and Methods were exploited to investigate the inhibitory effectiveness of the EtOAc layer of PEP on tumor growth in vivo. The results in Figure 4A demonstrated that the oral consumption of the EtOAc layer affected slightly the body weight of mice. The effects were more obvious in mice fed with 100 mg/kg EtOAc layer. As indicated in Figure 4B,C, the volume and weight of T24 tumors of the EtOAc layer-treated group (2, 5, 10, and 100 mg/kg) grew at a slower rate than those of the untreated tumors. On week 10, the tumor volumes of the control group had increased significantly to 600 mm^3^ on average, which is the endpoint of the animal protocol, whereas the tumor volumes of 5 and 10 mg/kg of EtOAc layer-fed animals had only reached 200 mm^3^ on average, respectively. The tumor weight of the control group increased to 456.5 mg on average while those of 5, 10, and 100 mg/kg EtOAc layer-fed mice decreased dramatically to 94.6, 94.9, and 39.4 mg, on average. The inhibitory effects on the tumor volume/weight were much higher than the body weight, suggesting that the decreased tumor volume/weight did not attribute to the body weight loss. Although EtOAc administration might impact the body weight, hematoxylin/eosin (H/E) staining of liver specimens showed that EtOAc layer demonstrated no lesion to the liver. The H/E stainings of the liver specimens were presented in Supplementary Figure S4.

To observe the impacts of the EtOAc layer on the xenografted tumors, T24 xenografted tumors, treated with H_2_O (vehicle), 2 mg/kg, 5 mg/kg, or 10 mg/kg of the EtOAc layer, were dissected from nude mice for histological examination. The hematoxylin/eosin (H/E) staining of H_2_O-treated tumors revealed large neoplastic areas while the 5 and 10 mg/kg treated counterparts showed less neoplastic areas comparatively (Figure 4B). Furthermore, the TUNEL assay was performed to evaluate if apoptosis was induced in treated xenografted tumors. The results in Figure 4C demonstrated that the amount of TUNEL-positive T24 cells had increased obviously in tumors treated with the EtOAc layer (2, 5, 10, and 100 mg/kg) after 10 weeks as compared to that of the control groups, suggesting that the apoptosis was evoked in treated xenografted tumors. The above results indicated that the apoptosis could be induced in UBUC in vivo, which was in line with the findings observed in UBUC cells.

## 4. Discussion

In this study, we found that the ethanol extract isolated from pomegranate peels exhibited a better inhibitory activity comparatively T24 and J82 cells than that of pulps. Fractionation of PEP with increasing polarity showed that the EtOAc layer of PEP had the best anti-cancer efficacy. Among 8 collecting parts of PEP fractionated with the Diaion HP-20 column chromatography, the PEPE2 fraction demonstrated the best inhibitory activity on UBUC cells along with the least influence on normal-like urothelial E7 cells. Annexin V/PI and western immunoblotting analyses demonstrated that PEPE2 treatment evoked UBUC cell apoptosis through stimulation of the death receptor pathway, the mitochondrial pathway, and ER stress. The suggested apoptotic pathway evoked by PEPE2 was shown in Figure 3E. The mitochondrial pathway starts with the increased permeabilization of the mitochondrial outer membrane, causing the rupturing of the outer membrane. The outer membrane rupturing the release of cytochrome c, apoptosis-inducing factor (AIF), endonuclease G, and Smac/DIABLO (second mitochondria-derived activator of caspases/direct IAP-associated binding protein with low pI) [16,17,18]. Cytochrome c along with apoptosis protease activating factor (APAF-1) and pro-caspase 9 form an apoptosome which activates caspase 9. Caspase 9, in turn, activates the effector caspases which orchestrates the progression of apoptosis [19]. AIF and endonuclease G participate in DNA fragmentation and subsequent chromosomal condensation, which is typical phenomena of apoptosis [17,18]. Smac/DIABLO can antagonize IAP (inhibitor of apoptosis protein) to promote caspase activation [20]. In addition to our proposed apoptotic mechanism, pomegranate peels might impair the release of cytochrome c, apoptosis-inducing factor (AIF), endonuclease G and Smac/DIABLO to interfere with the activation effector caspases. Taken together, our results implied that the components present in pomegranate peels demonstrated the inhibitory activity against UBUC cells and owned much better intervention capability than those of the pulps. The anti-cancer efficacy of PEP was further confirmed in the xenografted mice. It was observed that oral administration of the EtOAc layer of PEP to nude mice implanted with T24 cells caused a significant reduction in tumor growth. Besides, the TUNEL-positive cells were extensively observed in EtOAc layer-treated T24 tumors, suggesting cell apoptosis was evoked in extract-treated tumors.

Most of the chemopreventive/chemotherapeutic cancer studies of pomegranate fruits focus on the juice or various extracts prepared from the juice [1]. Very few documented findings emphasize the medicinal value of the pomegranate peels. The studies carried out by Zahin et al. (2014) showed that A549 and H1299 lung cancer cell lines demonstrate the comparable susceptibility to punicalagin and ellagic acid, the main ingredients of pomegranate peels [21]. In addition, treatment of the methanol extract of pomegranate pericarps (PME) results in the significant dose-responsive inhibition on cell proliferation in MCF7 cell lines that are positive for estrogen receptors (ER) while showing no impacts on that of ER^−^ MDAMB-231 cells. PME also reduces the expression of estrogen-responsive genes such as ERα, pS2, and PR in MCF7 cells. Examination on the PME estrogenicity indicated that there are no obvious differences in the uterus weight and the proliferation of uterine endometrium between PME- and vehicle-treated 17 β-estradiol-evoked ovariectomized mice, implying that PME is a selective estrogen receptor modulator [22]. Consistent with the above findings, Dikmen et al. (2011) also observed that the administration of pomegranate fruit peels (PPE) inhibits MCF7 cell proliferation and induces cancer cell apoptosis [23].

In addition to the above cancer cells, Asmaa et al. (2015) reported that the treatment of 80% ethanol extract of pomegranate peels (PGPE) mainly evokes G_2_/M cell cycle arrest to inhibit chronic myeloid leukemia K562 cells [24]. The compounds present in pomegranate peels possess inhibitory activities against prostate cancer. The effects of pomegranate peel extract (PoPx) on prostate cancer cell lines examined by Deng et al. (2017) demonstrated that PoPx incubation can induce apoptosis by the loss of mitochondrial transmembrane potential (Δym), the increased reactive oxygen species (ROS) as well as the augmented Bax/Bcl-2. Furthermore, they reported that PoPx treatment can retard migration/invasion likely through the down-regulation of matrix metallopeptidase (MMP) 2/MMP9 and the up-regulation of Metallopeptidase Inhibitor 2 (TIMP2) [25]. Gou et al. (2016) indicated that ellagic acid purified from pomegranate peels provokes Hela cell apoptosis by increasing the IGFBP7 expression level [26]. Song et al. (2016) found that treatment of polyphenol of pomegranate peels can induce HepG2 cells apoptosis by increasing cytochrome c amount, p53 expression level, Bax/Bcl-2 and caspase-3/9 activities. It can also arrest HepG2 cell at S-phase [27]. Most of the above-mentioned literature with regard to pomegranate peels focuses on the investigations of single cancer cell line. However, in this study, two cancer cell lines and animal studies were implemented for examination.

Besides anti-cancer activities, the pomegranate peel is demonstrated to have activities for other ailments. Due to its abundant phenolics and flavonoids, the pomegranate peel is indicated to possess high anti-oxidant activities [28]. Morzelle et al. demonstrated that the administration of pomegranate peel extract (PPE) decreases amyloid plaque density, augments the expression of neurotrophin BDNF, and reduces TNF-α production in mice infused with amyloid-β peptide. Their results imply that treatment of PPE provides neuroprotective effectiveness to the neurodegeneration provoked by infusion with amyloid-β peptide in mice [29]. PPE is also shown to alleviate the hepatic pathology, body weight, liver enzymes, and retard lipogenesis. It can enhance the cellular redox status in the liver tissues of rats with a non-alcoholic fatty liver disease to reduce oxidative damage [30]. Pomegranate peel extract is also showed to reduce significantly fasting blood glucose in type 2 diabetic mice, suggesting that it might be recommended for the management of type 2 diabetes [31]. Although a variety of studies related to the therapeutic effects of pomegranate peel have been reported, there are no clinical trial results.

Most of the UBUCs are papillary/non-invasive, or papillary/superficially invasive lesions. The lesions are normally treated by curettage but the local recurrence is often observed in papillary UBUCs, even followed by lethal distal spreading. The results of our studies implicated that the pomegranate peel extract may be a potential chemopreventive product for lowering the possibility of UBUC recurrence. Pomegranate peels cover the whole fruits and are handy to be separated from the fruit bodies, thus, it is easier to obtain than pulps. Furthermore, our results provide the likelihood that the traditionally non-edible pomegranate peel waste is re-utilized to make an affordable and promising chemopreventive product.

## Supplementary Materials

The following are available online at http://www.mdpi.com/2072-6643/10/5/543/s1. Supplementary document: legends for supplementary figure, Figure S1: The inhibitory activities of peel and pulp of pomegranate fruit. T24 (**A**) or J82 (**B**) cells were used to examine the inhibitory activities. PEP2 and PEP3 fractions from the EtOAc layer were examined for the toxicity to normal-like E7 cells (**C**), Figure S2: The HPLC profiles of EtOAc layer of PEP and PEPE2. (**A**) The HPLC profile of EtOAc layer of PEP. (**B**) The profile of PEPE2, Figure S3: The molecular mechanisms of apoptotic pathway evoked in PEPE2-incubated UBUC J82 cells. (**A**) pro-/cleaved caspase-8, DR4 and DR5, (**B**) pro-/cleaved caspase-9, Bax and Bcl-2, (**C**) Bip, VCP and pro- caspase-12 in PEPE2-incubated J82 cells. The immunoblot in each figure was the representative result of at least three independent experiments. The diagram (ratio (mean ± SD)) under each immunoblot indicated the ratio of normalized protein intensity (observed protein/actin) of PEPE2-treated cells at indicated time interval divided by that at 0-h time point. * *p* ≤ 0.05, ** *p* ≤ 0.01, *** *p* ≤ 0.001, Figure S4: liver specimens collected at 10th week from non-fed- and EtOAc layer-fed xenografted mice.
