# Supplementary material for: Deciphering the Molecular Mechanism Underlying the Inhibitory Efficacy of Taiwanese Local Pomegranate Peels against Urinary Bladder Urothelial Carcinoma
Source: Nutrients. 2018 Apr 27;10(5):543. doi: 10.3390/nu10050543 (PMC5986423; doi:10.3390/nu10050543)
Supplement: Supplementary file 1 [file nutrients-10-00543-s001.zip › Supplemenatry Figure.pptx]

## Slide 1
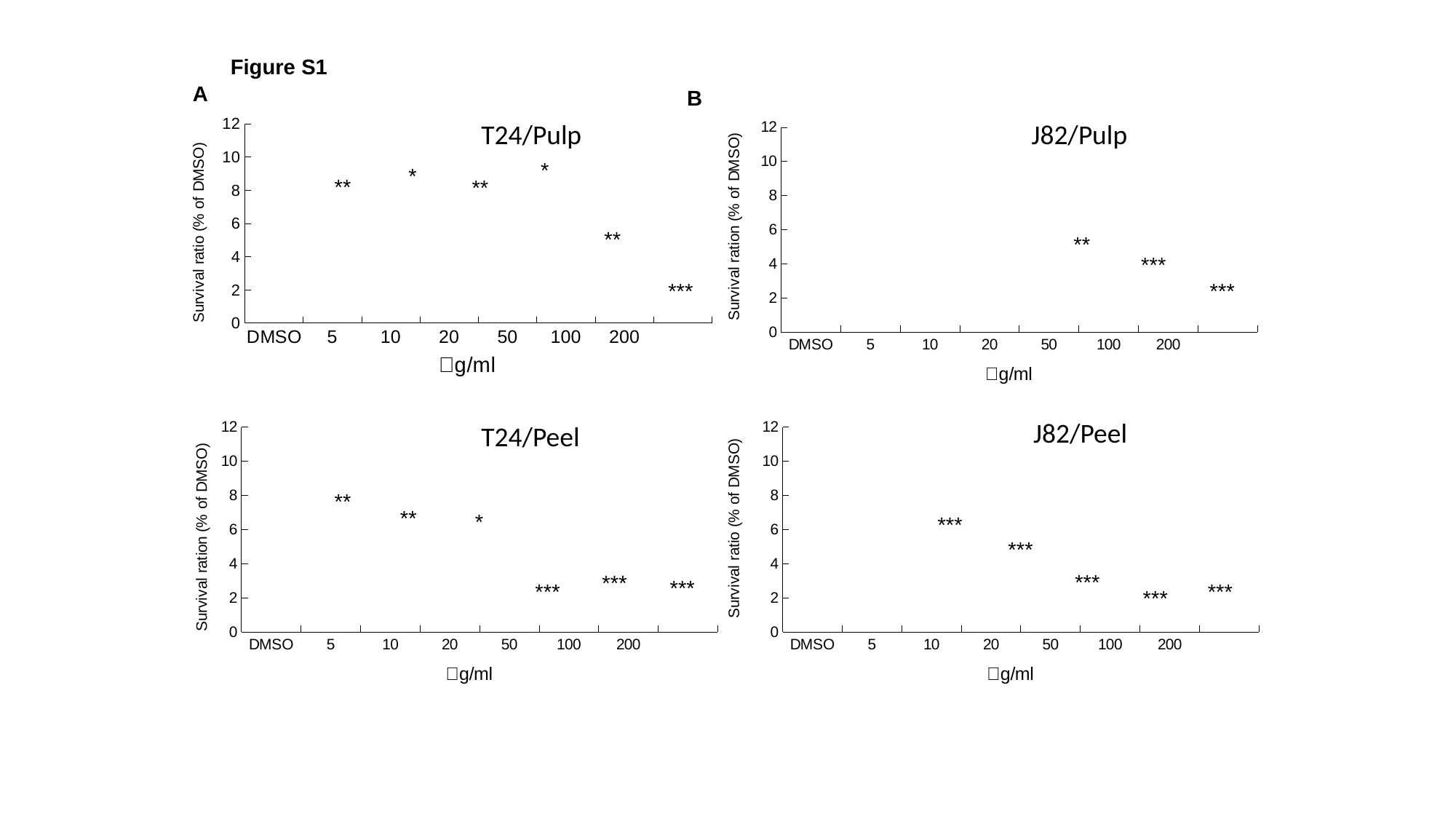

Figure S1
A
B
### Chart
| Category | |
|---|---|
| DMSO | 100.0 |
| 5 | 74.12008281573499 |
| 10 | 80.07246376811594 |
| 20 | 73.65424430641822 |
| 50 | 83.90269151138718 |
| 100 | 43.16770186335404 |
| 200 | 14.648033126293996 |
### Chart
| Category | |
|---|---|
| DMSO | 100.0 |
| 5 | 100.31746031746032 |
| 10 | 111.45299145299144 |
| 20 | 87.86324786324785 |
| 50 | 51.599511599511594 |
| 100 | 38.046398046398046 |
| 200 | 21.61172161172161 |T24/Pulp
J82/Pulp
*
*
**
**
**
**
***
***
***
### Chart
| Category | |
|---|---|
| DMSO | 100.0 |
| 5 | 69.63606735469853 |
| 10 | 57.84899511135253 |
| 20 | 44.43237370994026 |
| 50 | 18.631178707224333 |
| 100 | 22.813688212927758 |
| 200 | 21.89027702335687 |
### Chart
| Category | |
|---|---|
| DMSO | 100.0 |
| 5 | 79.8082684242061 |
| 10 | 57.04014379868185 |
| 20 | 44.06830437387657 |
| 50 | 26.06351108448172 |
| 100 | 17.0760934691432 |
| 200 | 18.813660874775312 |J82/Peel
T24/Peel
**
**
*
***
***
***
***
***
***
***
***

## Slide 2
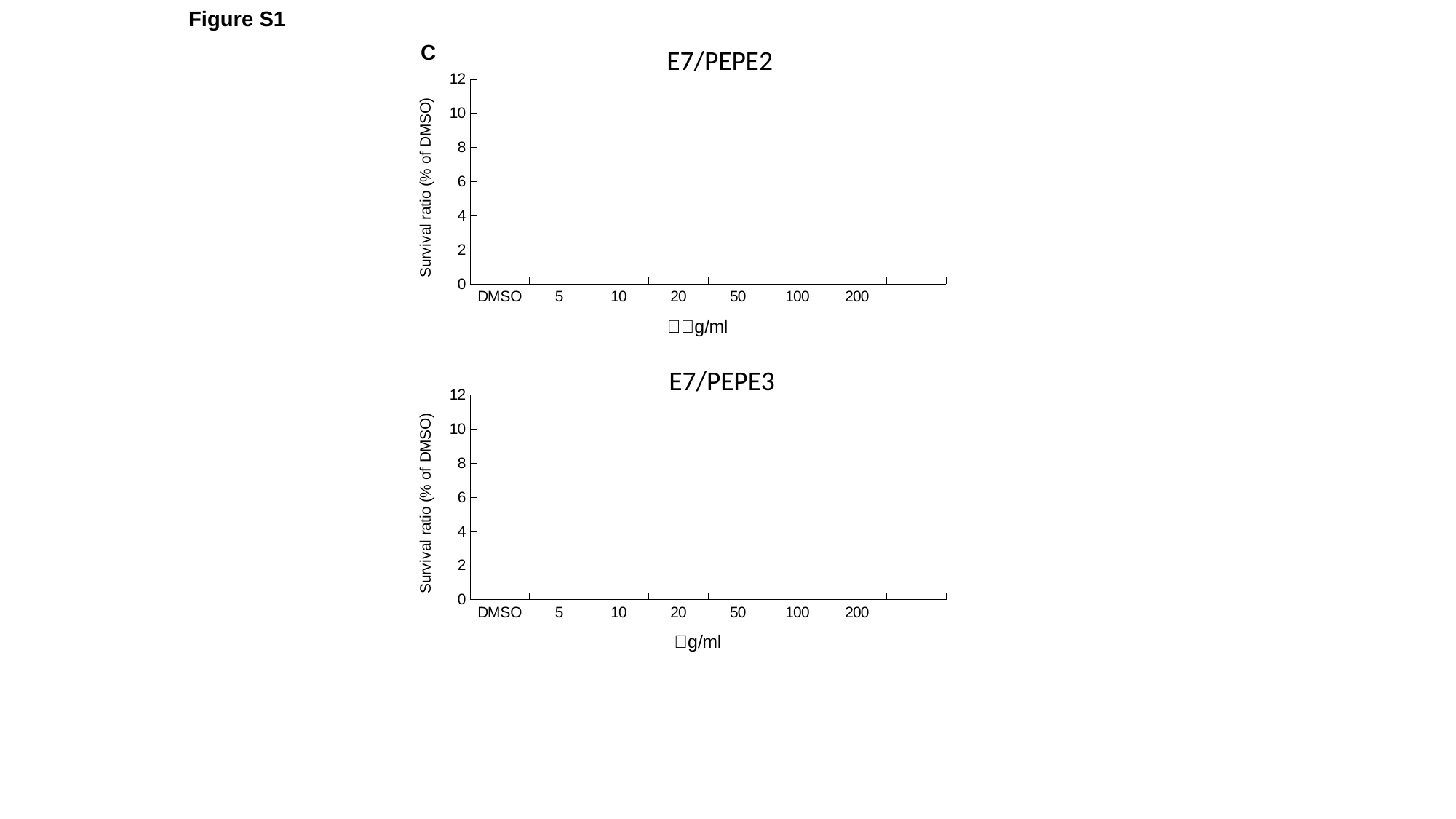

Figure S1
C
E7/PEPE2
### Chart
| Category | |
|---|---|
| DMSO | 100.0 |
| 5 | 85.7546145494028 |
| 10 | 87.79587404994571 |
| 20 | 102.62757871878392 |
| 50 | 101.54180238870792 |
| 100 | 85.32030401737241 |
| 200 | 56.48208469055374 |E7/PEPE3
### Chart
| Category | |
|---|---|
| DMSO | 100.0 |
| 5 | 95.4232283464567 |
| 10 | 86.13845144356956 |
| 20 | 90.45275590551182 |
| 50 | 87.02427821522309 |
| 100 | 85.99081364829397 |
| 200 | 88.41863517060368 |

## Slide 3
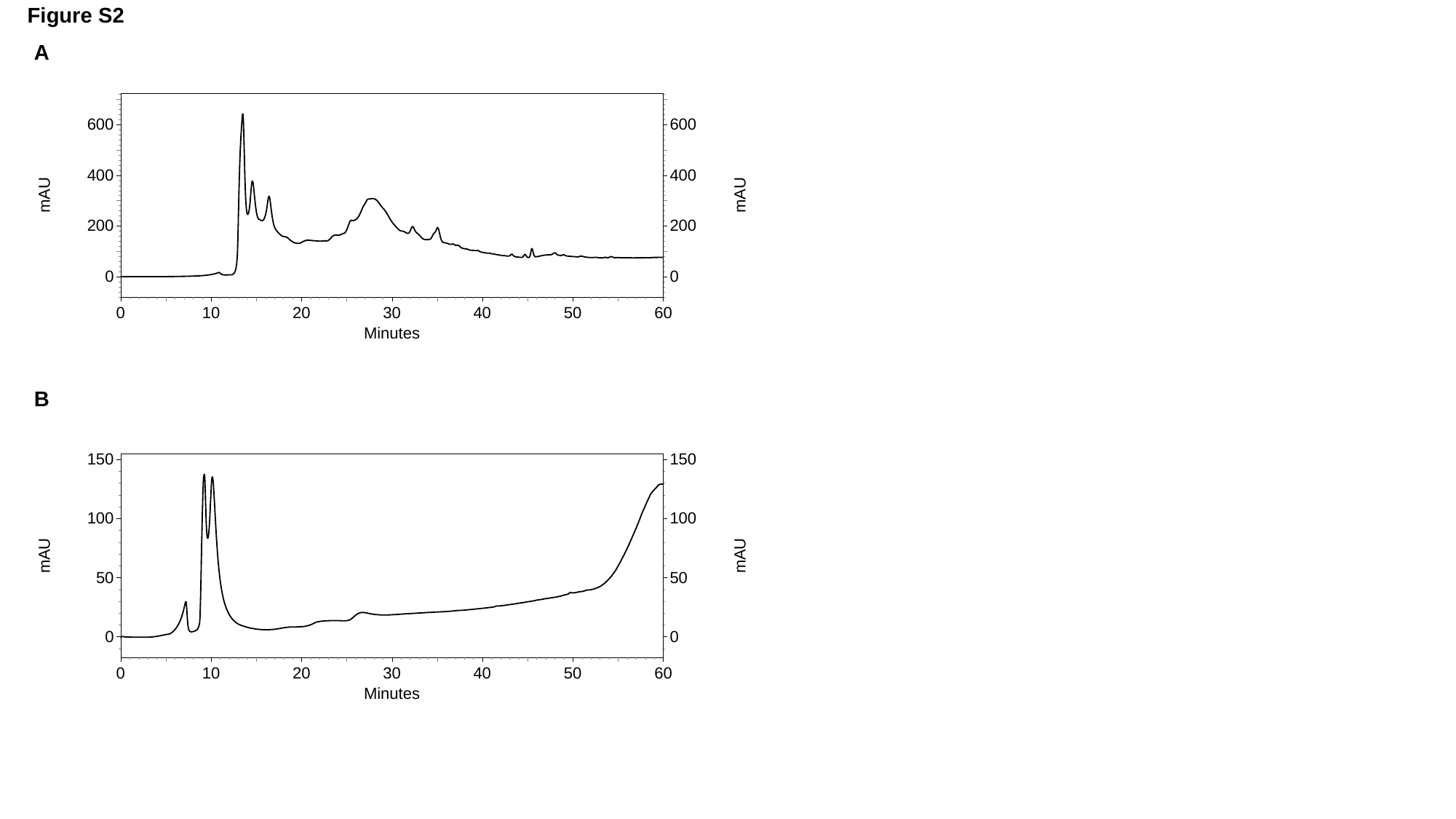

Figure S2
A
B

## Slide 4
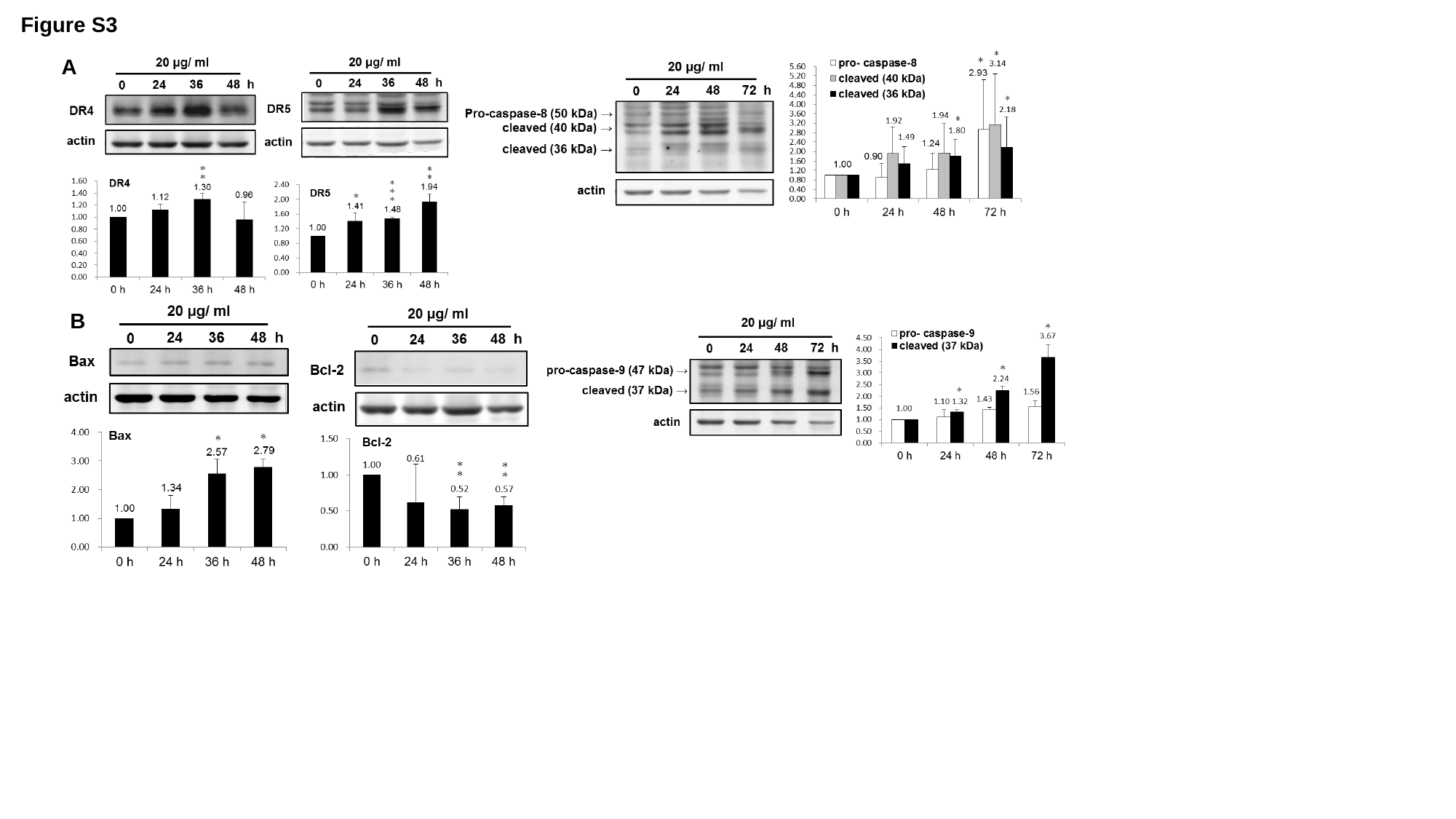

Figure S3
A
B

## Slide 5
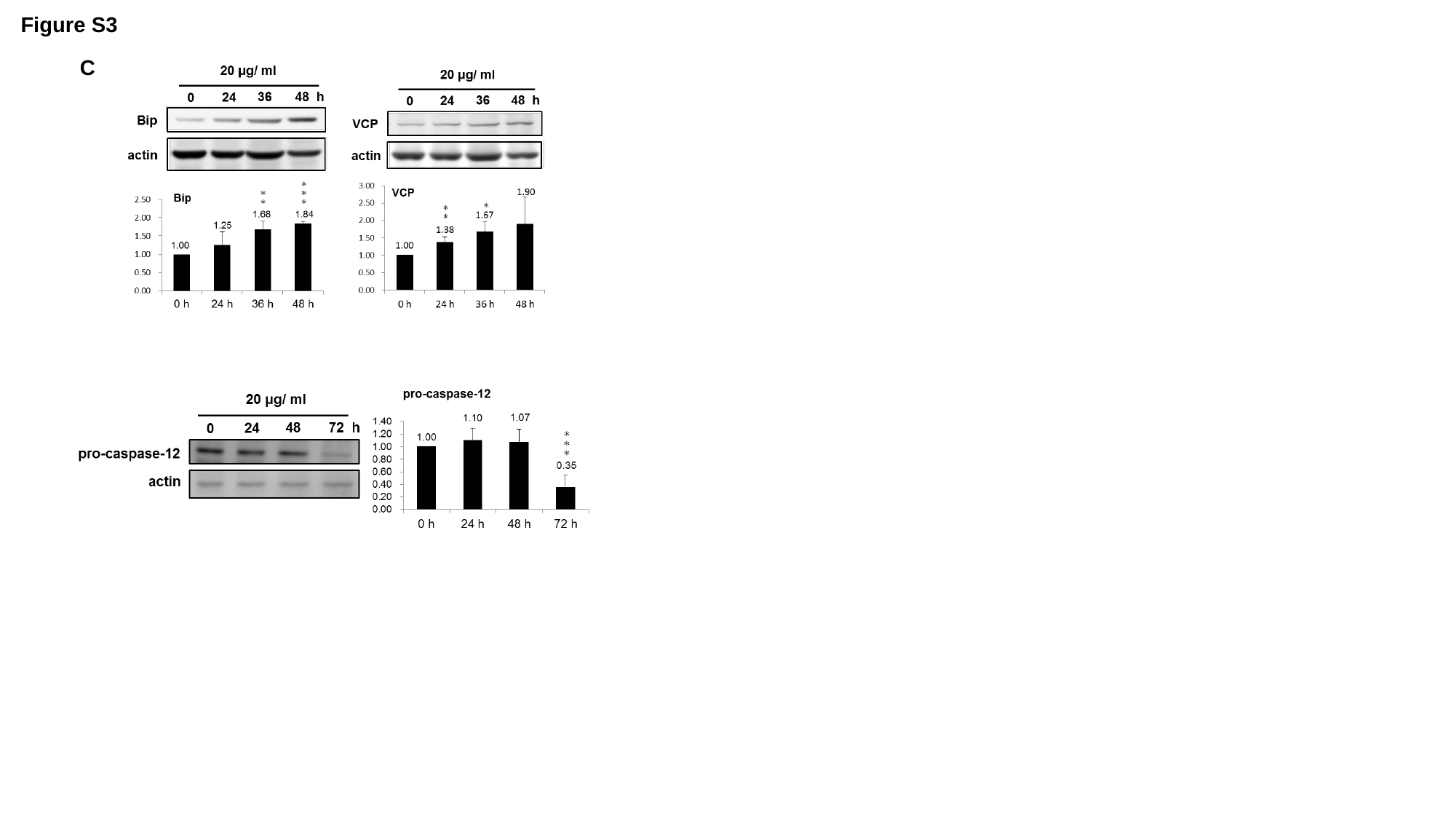

Figure S3
C

## Slide 6
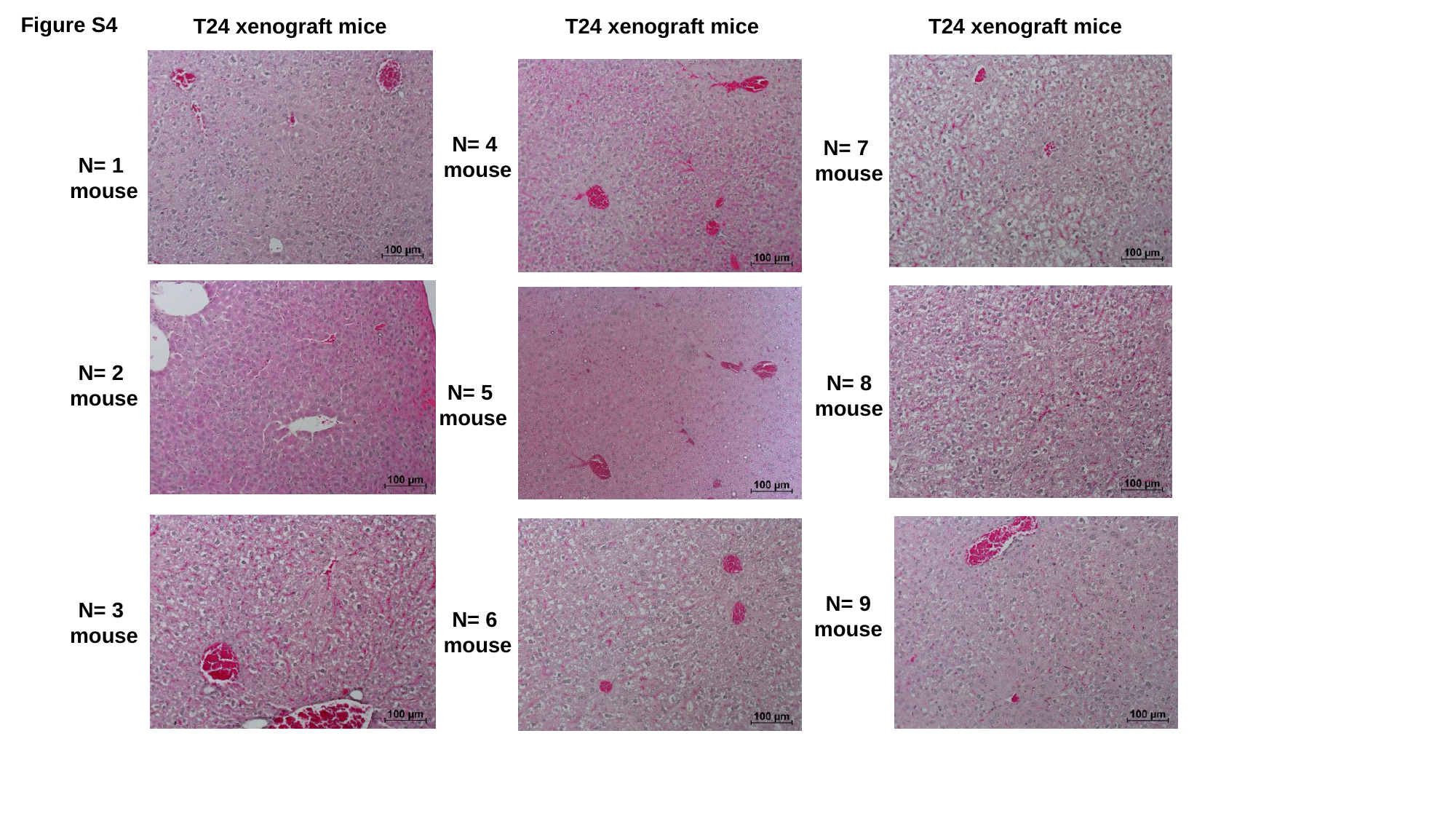

Figure S4
T24 xenograft mice
T24 xenograft mice
T24 xenograft mice
N= 4
mouse
N= 7
mouse
N= 1
mouse
N= 2
mouse
N= 8
mouse
N= 5
mouse
N= 9
mouse
N= 3
mouse
N= 6
mouse

## Slide 7
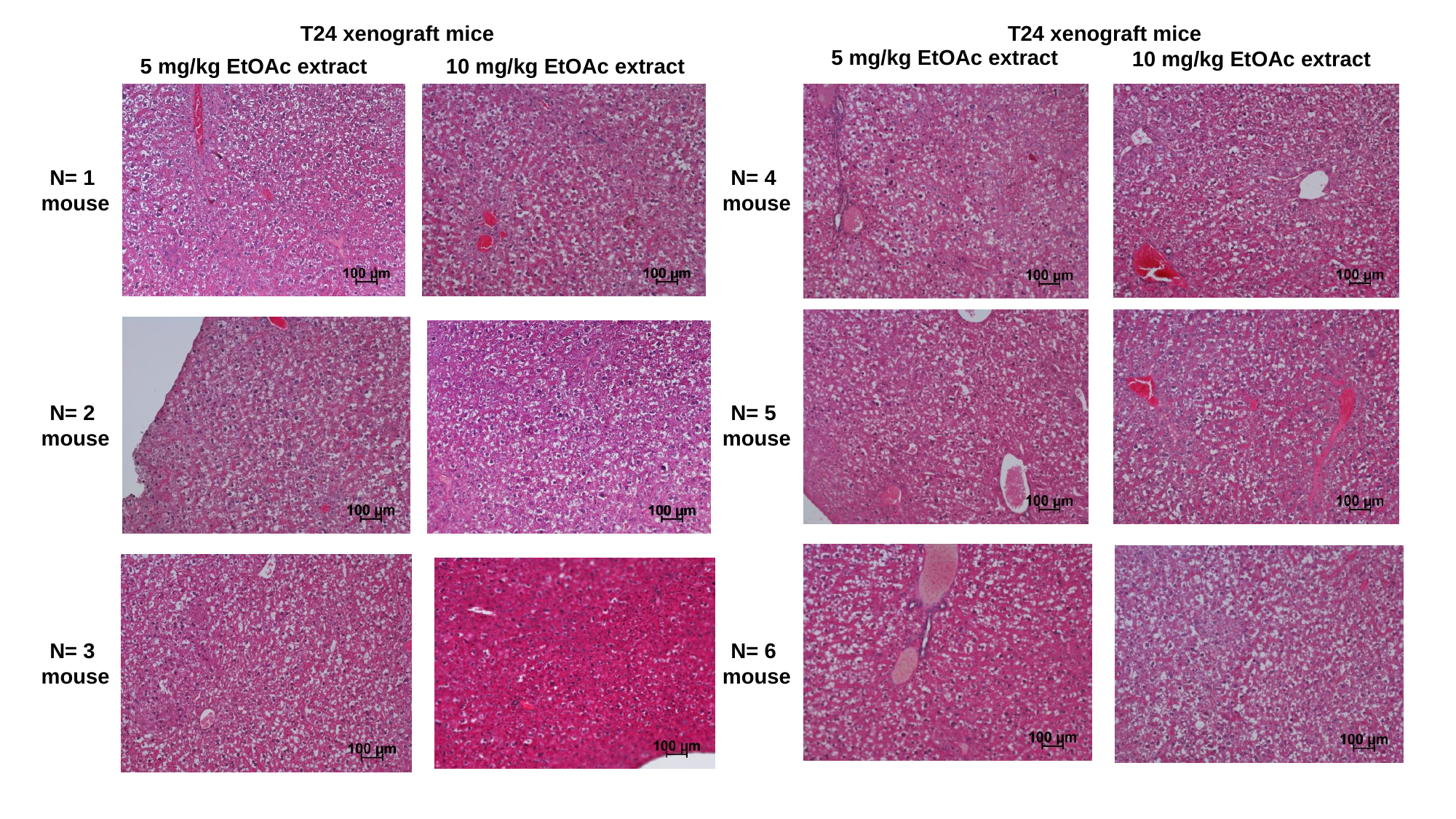

T24 xenograft mice
T24 xenograft mice
5 mg/kg EtOAc extract
10 mg/kg EtOAc extract
5 mg/kg EtOAc extract
10 mg/kg EtOAc extract
N= 1
mouse
N= 4
mouse
N= 2
mouse
N= 5
mouse
N= 3
mouse
N= 6
mouse

## Slide 8
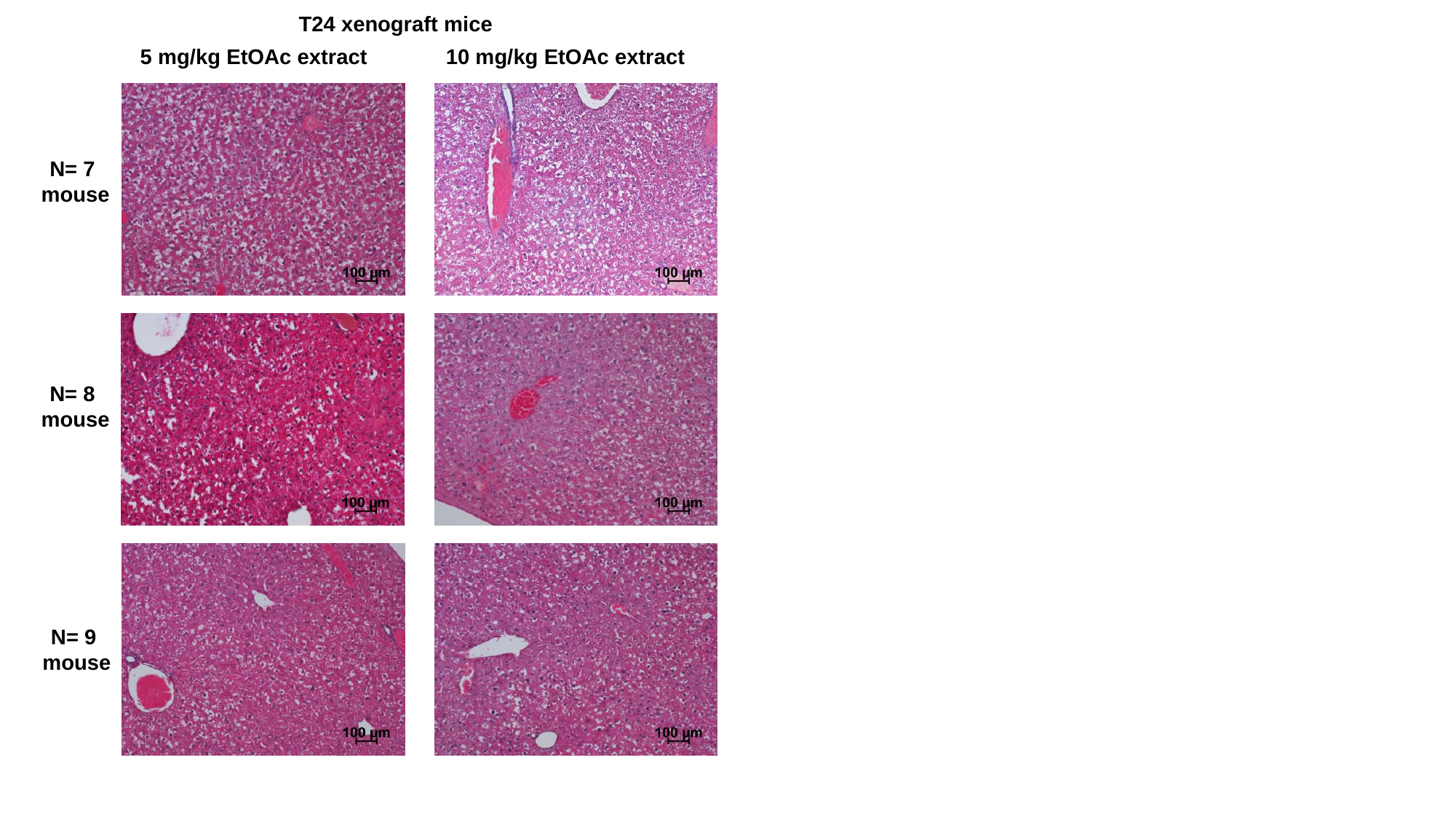

T24 xenograft mice
5 mg/kg EtOAc extract
10 mg/kg EtOAc extract
N= 7
mouse
N= 8
mouse
N= 9
mouse
