# Supplementary material for: Deciphering the Molecular Mechanism Underlying the Inhibitory Efficacy of Taiwanese Local Pomegranate Peels against Urinary Bladder Urothelial Carcinoma
Source: Nutrients. 2018 Apr 27;10(5):543. doi: 10.3390/nu10050543 (PMC5986423; doi:10.3390/nu10050543)
Supplement: Supplementary file 1 [file nutrients-10-00543-s001.zip › supporting information.docx]

**Legends for supplementary figure**

**Figure S1.** The inhibitory activities of peel and pulp of pomegranate fruit. T24 (A) or J82 (B) cells were used to examine the inhibitory activities. PEP2 and PEP3 fractions from the EtOAc layer were examined for the toxicity to normal-like E7 cells (C).

**Figure S2.** The HPLC profiles of EtOAc layer of PEP and PEPE2. (A) The HPLC profile of EtOAc layer of PEP. (B) The profile of PEPE2.

**Figure S3.** The molecular mechanisms of apoptotic pathway evoked in PEPE2-incubated UBUC J82 cells. (A) pro-/cleaved caspase-8, DR4 and DR5, (B) pro-/cleaved caspase-9, Bax and Bcl-2, (C) Bip, VCP and pro- caspase-12 in PEPE2-incubated J82 cells. The immunoblot in each figure was the representative result of at least three independent experiments. The diagram (ratio [mean±SD]) under each immunoblot indicated the ratio of normalized protein intensity (observed protein/actin) of PEPE2-treated cells at indicated time interval divided by that at 0-hour time point.**P* ≤ 0.05, ***P* ≤ 0.01, ****P* ≤ 0.001.

**Figure S4.** Liver specimens collected at 10th week from non-fed- and EtOAc layer-fed xenografted mice.
